# Society Meetings

**Published:** 1892-10

**Authors:** 


The American Association of Obstetricians and Gynecologists held one of the most interesting meetings in its history in the City of St. Louis, September 20, 21, and 22, 1892. Its members were cordially received by the profession of St. Louis, and generously entertained in various ways. As the Association does not accept of hospitalities, it was obliged to decline the proffer of any general reception.

The officers elected for the ensuing year are: *President*, Lewis S. McMurtry, M. D., Louisville; *Vice-Presidents*, Edward J. Ill, M. D., Newark, N. J., and Howard W. Longyear, M. D., Detroit; *Secretary*, William Warren Potter, M. D., Buffalo; *Treasurer*, X. O. Werder, M. D., Pittsburg. *Executive Council*, Charles A. L. Reed, M. D., Cincinnati; Geo. H. Rohé, M. D., Catonsville; James F. W. Ross, M. D., Toronto; William Wotkyns Seymour, M. D. Troy; and Donnel Hughes, M. D., Philadelphia.

The following-named physicians were elected to fellowships: *Ordinary*—W. E. Ashton, Philadelphia; F. Blume, Pittsburg; A. H. Cordier, Kansas City; E. W. Cushing, Boston; W. B. Dewees, Salina, Kans.; L. H. Dunning, Indianapolis; John M. Duff, Pittsburg; W. B. Dorsett, St. Louis; Geo. F. Hulbert, St. Louis; B. M. Hypes, St. Louis; Willis P. King, Kansas City; James T. Jelks, Hot Springs; A. B. Miller, Macon City, Mo.; M. Rosenwasser, Cleveland. *Honorary*—L. Ch. Boislinere, St. Louis.

The next meeting will be held in Detroit on Thursday, Friday, and Saturday, June 1, 2, and 3, 1893.

